# Inheritance and Genetic Mapping of the Reduced Height (*Rht18*) Gene in Wheat

**DOI:** 10.3390/plants7030058

**Published:** 2018-07-15

**Authors:** Nathan P. Grant, Amita Mohan, Devinder Sandhu, Kulvinder S. Gill

**Affiliations:** 1 Department of Crop and Soil Sciences, Washington State University—Pullman, 277 Johnson Hall, PO Box 646420, Pullman, WA 99164-6420, USA; ngrant@wsu.edu (N.P.G); amitamohan@wsu.edu (A.M.); 2 USDA-ARS Salinity Lab., 450 W. Big Springs Rd., Riverside, CA 92507, USA

**Keywords:** *Rht18*, reduced height, wheat, semi-dwarf, linkage map

## Abstract

Short-statured plants revolutionized agriculture during the 1960s due to their ability to resist lodging, increased their response to fertilizers, and improved partitioning of assimilates which led to yield gains. Of more than 21 reduced-height (*Rht*) genes reported in wheat, only three—*Rht-B1b*, *Rht-D1b*, and *Rht8*—were extensively used in wheat breeding programs. The remaining reduced height mutants have not been utilized in breeding programs due to the lack of characterization. In the present study, we determined the inheritance of *Rht18* and developed a genetic linkage map of the region containing *Rht18*. The height distribution of the F_2_ population was skewed towards the mutant parent, indicating that the dwarf allele (*Rht18*) is semi-dominant over the tall allele (*rht18*). *Rht18* was mapped on chromosome *6A* between markers barc146 and cfd190 with a genetic distance of 26.2 and 17.3 cM, respectively. In addition to plant height, agronomically important traits, like awns and tiller numbers, were also studied in the bi-parental population. Although the average tiller number was very similar in both parents, the F_2_ population displayed a normal distribution for tiller number with the majority of plants having phenotype similar to the parents. Transgressive segregation was observed for plant height and tiller number in F_2_ population. This study enabled us to select a semi-dwarf line with superior agronomic characteristics that could be utilized in a breeding program. The identification of SSRs associated with *Rht18* may improve breeders’ effectiveness in selecting desired semi-dwarf lines for developing new wheat cultivars.

## 1. Introduction

The Green Revolution, in the mid-twentieth century, brought about advancements in agriculture that are still in practice to date. The introduction of semi-dwarf varieties that are more responsive to changing agriculture practices like response to fertilizers was pivotal in bringing the green revolution by increasing cereal production to meet the population demands particularly in developing countries like China, India, Brazil, and Egypt [1]. Two genotypes, Norin10 {*Rht1* (*Rht-B1b*) and *Rht2* (*Rht-D1b*)} and Akakomugi (*Rht8*), were first incorporated into breeding programs to introduce the semi-dwarf genes in wheat cultivars in the United States and Italy [2,3].

The development of semi-dwarf cultivars can be attributed to a shorter yet stronger culm that accommodates high yields and prevents lodging [4,5]. Of the 21 wheat mutants reported to be associated with height reduction, only *Rht-B1b* and/or *Rht-D1b*, *Rht8* and *Rht12* have been characterized in detail [6,7]. *Rht-B1* and *Rht-D1* are two homoeologous genes present on B and D genomes in hexaploid wheat that code for DELLA proteins, which suppress gibberellin (GA)-responsive growth [8]. Normally, GA regulates binding of the GA insensitive dwarf 1 (GID1) receptor protein with DELLA proteins and promotes their degradation. Mutant alleles, *Rht-B1b* and *Rht-D1b*, produce DELLA proteins that do not bind GID1 resulting in growth inhibition due to insensitivity to GA [8]. Similarly, modulation in GA synthesis or signaling is known to be involved in reducing plant height in different species. Studies in *Arabidopsis*, maize [6], rice [9,10], and barley [11], suggest that GA affects the inter-nodal elongation and thus alters plant height. 

Height reduction in present day cultivars of wheat is achieved mainly by *Rht-B1b* and/or *Rht-D1b*, accounting for ~95% of the cultivated wheat around the world [2]. Of the other 19 height mutants reported in wheat, only *Rht8* has been used in some European wheat cultivars. The rest have not been utilized either because of the lack of genetic characterization or mapping information. The limited genetic variability in semi-dwarf lines used in breeding programs is becoming a bottleneck for further wheat improvement, due to the association of some negative effects with the *Rht-B1b* and *Rht-D1b* genes, particularly under abiotic stresses or changing environmental conditions [12]. Currently used semi-dwarf wheat lines are defective from the perspective of GA, which plays an important role in the growth and development of the plant. These genotypes display a significant effect on early seedling growth. Specifically, coleoptile length, first leaf elongation, seedling emergence, and plant height reduction have been reported in the genotypes carrying *Rht-B1b* and *Rht-D1b* compared to tall parents [6,13]. The GA-responsive *Rht12* and *Rht13* were reported to reduce plant height with no adverse effect on the coleoptile and root trait during the seedling stage [14,15]. *Rht12* delayed ear emergence, reducing flag leaf length and grain size, while *Rht13* adversely affected the 1000 kernel weight and flag leaf length. Initially classified as GA-responsive, *Rht8* was reported to be involved in reduced sensitivity to brassinosteroids that resulted in reducing plant height [16]. The 17 other reduced-height mutants have not been fully characterized. *Rht18* was found to be GA-sensitive and was identified as a possible reduced height mutant candidate for future breeding programs [4,17]. In durum wheat, *Rht18* was previously mapped to the short arm of chromosome *6A* at the same locus as *Rht14* and *Rht16* [17,18]. Applications of exogenous gibberellins (GA_3_) restored plant height and other agronomic traits of *Rht18* dwarf lines to the wild-type levels, indicating that *Rht18* dwarf mutants are impaired in GA biosynthesis [19]. In this investigation, we have mapped *Rht18* to chromosome *6A* using a cross between a pre-green revolution tall line (Indian) devoid of any know height reducing genes and *Rht18* mutant Icaro. The transfer of the *Rht18* allele into bread wheat and the selection of potential semi-dwarf lines with good agronomic characteristics can be useful for wheat breeding programs. 

## 2. Results and Discussion

### 2.1. Plant Height of F_2_ and F_2:3_ Progenies

**Figure 1:**
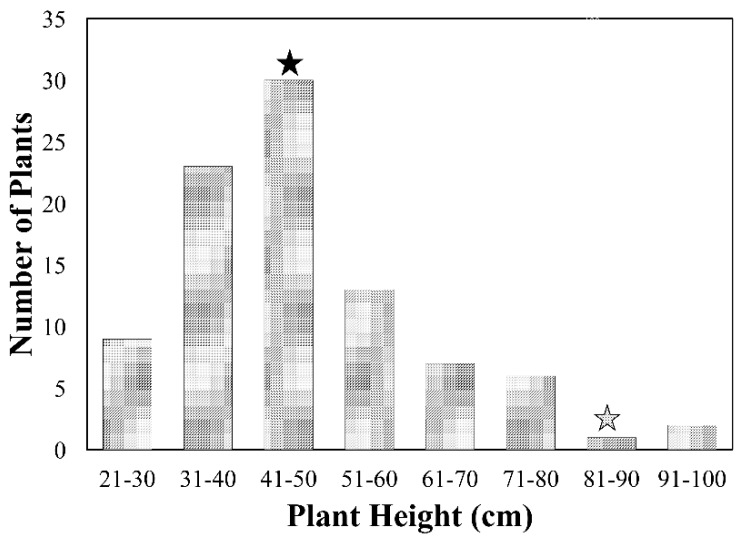
Height distribution in the F_2_ population. Plant height was grouped into 10 cm series. The star represents plant height of tall or dwarf mutant parent. The average plant height of Indian is recorded 86 cm (from eight plants) and Icaro as 44 cm (from six plants).

The plant height of the F_2_ population was recorded under controlled environmental conditions in a greenhouse along with parental lines Indian and Icaro. The height of the tall parent Indian and dwarf mutant parent Icaro averaged 86 ± 2.82 cm (Mean ± S.E.) and 44 ± 1.02 cm (Mean ± S.E.), respectively (Figure 1). Of the 94 F_2_ plants, approximately 55 were within 10 cm of the Icaro height range. Only four of the plants in the F_2_ population had a phenotype similar to Indian (86 ± 10 cm). Three of the originally-sown plants did not grow to maturity. This is expected as sterility is often associated with the incompatibility among the tetraploid and hexaploid crosses [20,21]. The F_2_ population had a height distribution skewed towards the parent Icaro (Figure 1). The skewed distribution towards reduced height parents was also reported in the *Rht3* F_2_ mapping population [22]. This distribution suggests that the mutant phenotype is dominant, as only a few plants had the tall phenotype. Interestingly, a few F_2_ plants were taller than the tall parent and many were shorter than the dwarf parent. The height distribution pattern suggests that additional modifier genes might be involved in regulating plant height. Plant height is known to be a complex trait regulated by interaction and interplay among major and minor genes [23]. The transgressive segregation observed for plant height might be due to epistatic gene actions [23]. Transgressive segregation was reported earlier in wheat for several agronomic traits, including plant height [24], grain yield and its components [25], heading date, and vernalization requirement [26]. In a previous study involving *Rht8*, transgressive segregants were observed for longer peduncles and grains per spike with no significant change in spike length, spikelet number, or number of fertile tillers [27]. Additionally, no significant effect was observed on roots, while a slight decrease in coleoptile length occurred. Partitioning of dry matter to ears was increased at anthesis, however, dry weight of stems and above-ground biomass, including ears, decreased [27].

**Figure 2:**
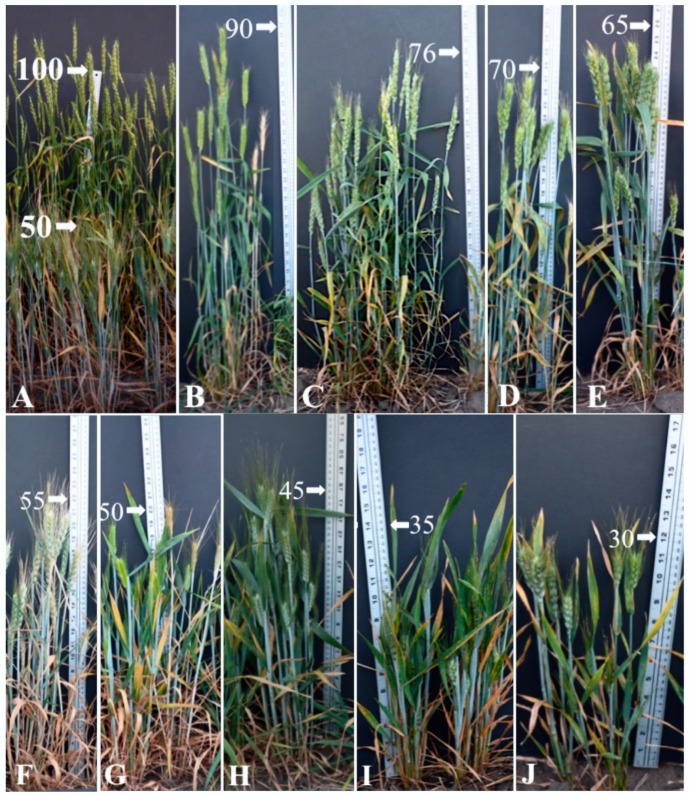
Plant height distribution among F_2:3_ families. (**A**) Indian and Icaro; and (**B**–**H**) different F_2:3_ families.

Forty seeds from each individual F_2_ plant representing the F_2:3_ progenies were sown in the field the following summer to evaluate the genotypes of the F_2_ plants, as it was difficult to classify plants into distinct categories in F_2_. The F_2:3_ population showed segregation for plant height (Figure 2), with 14 progenies classified as homozygous short, one as homozygous tall, and 54 were classified as heterozygous. Highly significant effects were found for the plant height (Table 1). For the F_2:3_ population the height was found to be on average taller than the F_2_, possibly due to the photoperiod effect in the field. As seen in the F_2_ generation, we observed some very dwarf and some very tall plants in F_2:3_ progenies (Figure 2), indicating the role of additional modifier genes in transgressive segregation. 

### 2.2. Spike Morphology

**Figure 3:**
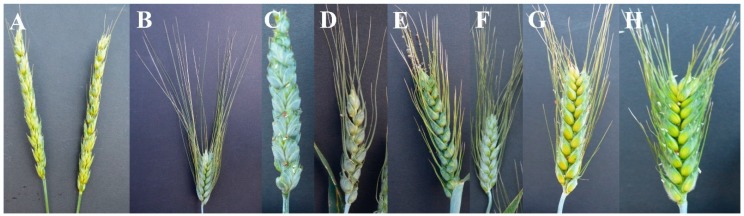
Spike morphology of parents and F_2:3_ families in the mapping population. (**A**) Indian; (**B**) Icaro; and (**C**–**H**) different F_2:3_ progenies.

Along with the plant height, the F_2_ population also segregated for awn-less/short or long, black awns. Among the parents, Indian spikes were awn-less and Icaro spikes bear long black awns (Figure 3). Among the F_2_ plants, 55 plants had awns and 36 plants were awn-less. We have also observed a difference in spike morphology among the F_2_ and F_2:3_ plants (Figure 3). The Indian spike is long and had loose spikelets, while the Icaro head is small with compact spikelets (Figure 3). We have observed plants with Indian-type heads with awns and Icaro-type heads without awns (Figure 3). 

### 2.3. Tiller Number

**Figure 4:**
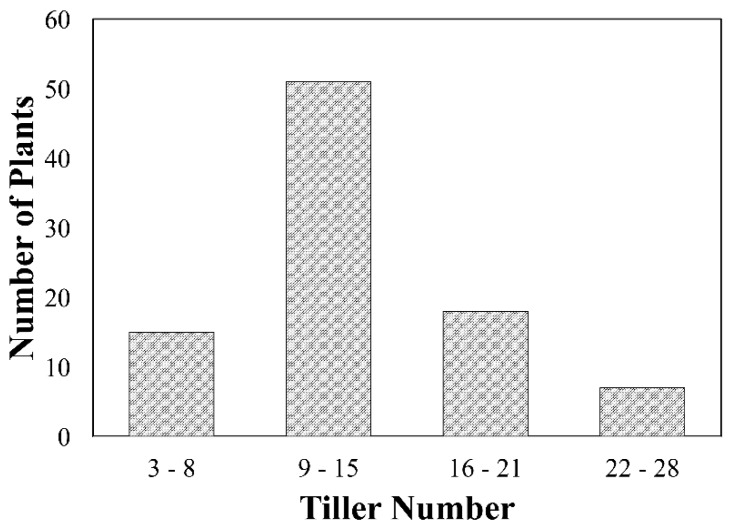
Tiller number distribution in the F_2_ population. The average number of tillers for parents Indian and Icaro were 11 and 12, respectively.

The F_2_ population displayed a range for the number of tillers per plant ranging from three tillers per plant to 28 tillers per plant (Figure 4). Fifty-five percent of plants have tiller numbers ranging from 9 to 15 per plant, resembling the average for both Indian and Icaro, which were approximately 11 and 12 tillers per plant, respectively. The highest tillering plants were usually dwarf and sterile or contained only a few seeds in a spike. This might be due to incompatibility between the two genotypes. The higher or lower number of tillers compared to the average of both the parents might be due to multigenic nature of the trait. Extreme dwarf plants were sterile and did not set seeds. Further, of the 91 F_2_ lines used for F_2:3_ field evaluation, only 75 plants produced seed. This is expected for a hexaploid and tetraploid cross due to pollen viability issues restricting the seed set [28]. Among the plants that set seeds, some had good seed sets while others only contributed a few per plant.

The variation observed in spike morphology was not associated with the height phenotype each plant displayed (data not shown). The seed weight did not correlate with plant height. Tiller number in F_2_ plants did not associate with the seed weight or number of seeds harvested at maturity (data not shown). The 100 seed weight for Indian and Icaro were 3.43 g and 3.69 g, respectively. Among the F_2:3_ families, the short families had an average 100 seed weight of 2.8 g while the tall families had 3.1 g. The height mutation in wheat is reported to have affected the seed weight compared to the tall counterpart. Of the studied reduced height mutants, *Rht12* reduces the grain weight more compared to *Rht-B1b*, *Rht-B1c*, and *Rht8* [29]. The reduction in grain weight might be due to the adverse effect of *Rht18* on grain size [27,30]. In fields conditions, the tiller number per plant was difficult to measure, hence, was not recorded for the F_2:3_ plants. The F_2:3_ families were also evaluated in the field for their agronomic characteristics to identify the agronomically important plant to be utilized in hexaploid wheat breeding. We have selected one line (line 29) based on plant height, stem strength, and spike morphology. More detailed agronomic and molecular analysis will be performed on the selected line to determine its suitability for utilization in a breeding program.

### 2.4. Genetic Mapping of Rht18

**Figure 5:**
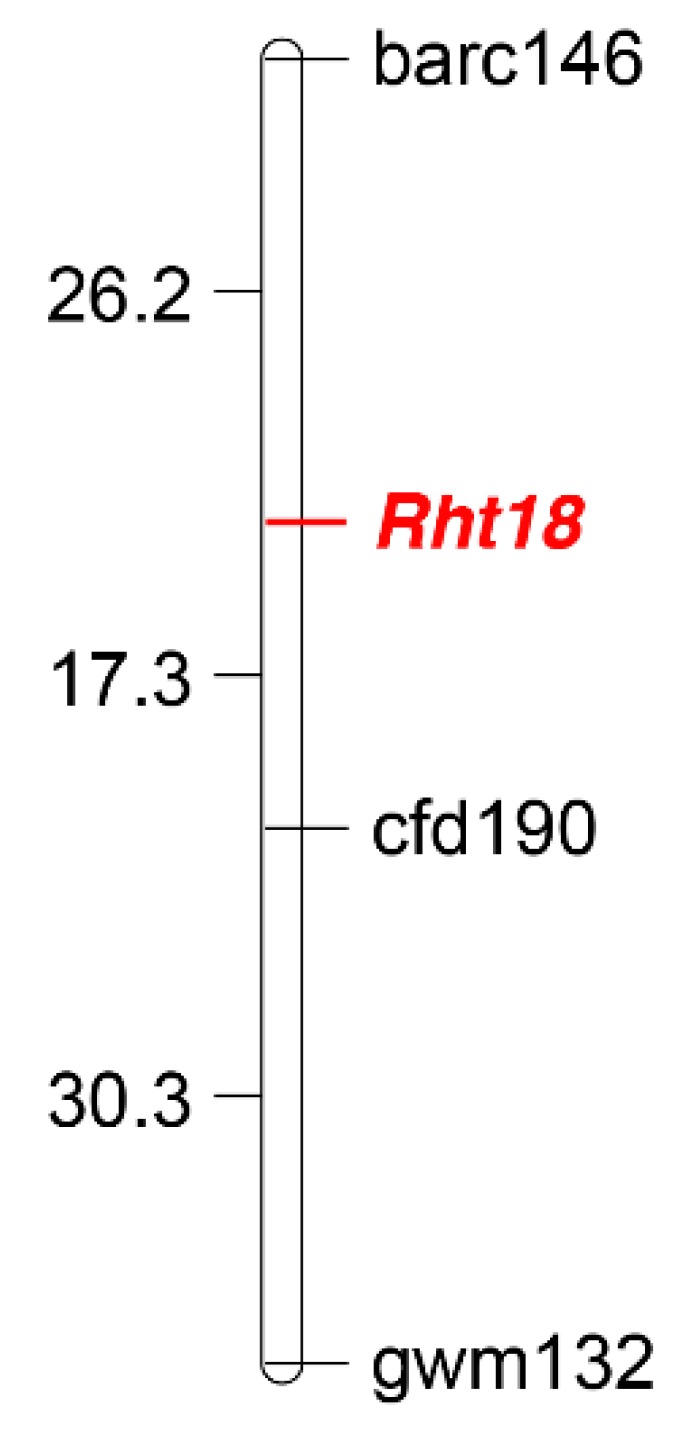
Genetic linkage map showing the position of *Rht18* on chromosome *6A*. Genetic distances are shown in centiMorgans (cM).

In order to map the gene on a wheat chromosome, over 700 SSR markers [31] were used to screen parents Indian and Icaro. Of these, 154 markers showed polymorphism between the parents and were used to genotype the population. The *Rht18* gene was mapped to the short arm of chromosome *6A* and was flanked by barc146 and cfd190 (Figure 5). The SSR marker cfd190 was placed at a distance of 17.3 cM away from *Rht18*. Previously, barc003, a marker from the short arm of the chromosome *6A*, was mapped 25.1 cM away from *Rht18* in durum wheat [17,32]. Earlier, *Rht18* was mapped on chromosome *6A* between barc118 and IWA4371 using recombinant inbred lines (RILs) in durum wheat [18]. The mapping location of *Rht18* in our study is consistent with the previous map position [17,18]. Recently, several independent single nucleotide variants in the *GA2oxA9* gene located on chromosome 6A were associated with the *Rht18* mutant phenotype [33]. *GS20xA9* is predicted to encode GA 2-oxidase, which reduces the amount of bioactive GA (GA1). 

Reduced-height genes in wheat have been imperative to the agronomic success of the crop. The resulting yield increases have been credited to the improved structure of the plant that responded better to the agronomic practices in use today. The semi-dwarf phenotype increases resistance to lodging along with increasing the number of grains per plants. Incorporating additional reduced-height genes into breeding programs could help contribute to the diversification of the genotype. Considering climate change and the demand for food security, incorporating additional dwarfing genes into the germplasm and evaluating their agronomic worth might help to address the wheat productivity under a changing climate. As the photoperiod and the background of a genotype affect height, a marker close to the gene may assist in easy and precise selection of the locus. Thus, identification of SSR markers closer to the *Rht18* locus may assist breeders in early identification of dwarfing lines for breeding populations. Further, conducting the genomic and agronomic characterization of this mutant gene may become instrumental in developing a better dwarfing system in wheat. Additionally, we have identified a semi-dwarf line from F_2:3_ families with superior agronomic characters that might have potential to be used in wheat breeding to incorporate the gene into the hexaploid background of Pacific Northwest region.

## 3. Materials and Methods 

### 3.1. Plant Materials

The dwarf parent, Icaro (tetraploid; 4×) (*Rht18*; PI 503555), was originally derived in 1987 in Italy from fast-neutron treatment of cv. Anhinga (PI 428455). The tall line Indian (hexaploid; 6×) (CI 4489), was developed at the University of Idaho, Idaho before 1915. As the tall parent is released before the introduction of reduced height genes, we presumed that cv. Indian would be devoid of the *Rht18* allele in the background. Both the germplasms were procured from GRIN [34].

### 3.2. F_1_ and Plant Growing Conditions

The F_1_ produced by crossing Indian as the female parent and Icaro as the male parent was self-pollinated, and 120 F_2_ seeds were collected. The F_2_ mapping population was grown at the plant growth facility, Washington State University, under controlled conditions of 16 h days (22 °C) and 8 h (18 °C) nights. For ease of genotyping, 94 randomly-selected F_2_ plants were selected for further analysis. Forty seeds of each F_2_ plant were grown in three-foot rows at the Spillman Agronomy Farm, Pullman, WA for phenotypic screening. Four rows were planted in each plot with a row-to-row and plot-to-plot spacing of one foot. Each row represented progeny of a single F_2_ plant. The seeds were planted mechanically using four planter drills and the plants were grown until maturity using the standard regional agricultural practices with no irrigation.

### 3.3. Phenotypic Screening

The phenotypic data for height, awns, tiller number, and seed weight was collected for the F_2_ and F_2:3_ populations. The plant height was recorded at maturity to the nearest cm excluding the awns. The population was characterized into tall, intermediate, and dwarf based on the plant height at maturity. Tiller numbers were counted manually per plant and seed weight was measured for each individual plant.

### 3.4. DNA Isolation and Genotyping

Young leaf tissue of F_2_ plants was collected in 96-well DNA extraction plates. Four, 2-cm long leaf segments were clipped and lyophilize for three days. The lyophilized tissue was used for DNA isolation using a modified SDS extraction method [35]. The DNA was diluted to a final concentration of 25 ng/µL. Primer sequence information for simple sequence repeat (SSR) markers were obtained from GrainGenes website [36].

Over 700 SSR markers were first screened for polymorphism between the parental genotypes. The PCR was performed in 12 µL reaction volume containing 1× NEB reaction buffer, 200 µM of dNTPs, 2.5 mM MgCl_2_, 0.05 µM forward primer, 0.25 µM reverse primer, 0.2 µM M13 forward-labeled primer, and 1U homemade Taq polymerase. For multiplexing, the M13 sequence was fluorescently labeled separately with FAM, HEX, NED, and PET dyes. The amplification of SSR loci was performed using the protocol consisted of 94 °C/4 min for initial denaturation, followed by 37 cycles (94 °C/30 s, 60 °C/45 s, 72 °C/60 s), with final extension at 72 °C/10 min. The amplification products were separated using ABI DNA analyzer 3100 (Applied Biosystems Inc., Carlsbad, CA, USA). Alleles were sized relative to internal size standard (cassual445 labeled with Dy630) using GeneMarker software (Softgenetics, State College, PA, USA). MapMaker 2.0 was used to construct the genetic linkage map using the Kosambi mapping function [37,38].

## Figures and Tables

**Table 1:** Analysis of variance (ANOVA) of plant height for the F_2:3_ population.

Source	DF	SS	MS	F Value	Pr > F
**Model**	76	155,138.4	2041.29	14.49	<0.0001
**Error**	648	9131.879	140.91		
**Corrected total**	724	246,452.3			
